# The Rediscovery and Life History of the Enigmatic Weevil *Hypera libanotidis* (Coleoptera: Curculionidae): A Description of the Mature Larva and Pupa After More than a Century

**DOI:** 10.3390/insects17030248

**Published:** 2026-02-26

**Authors:** Jiří Skuhrovec, Rafał Gosik, Jiří Krátký, Valentin Szénási, Filip Trnka

**Affiliations:** 1 Group Function of Invertebrate and Plant Biodiversity in Agro-Ecosystems, Czech Agrifood Research Center, Prague 6-Ruzyně, 161 00 Prague, Czech Republic; jirislavskuhrovec@gmail.com; 2 Zoological Museum, Faculty of Biology and Biotechnology, Maria Curie-Skłodowska University, Akademicka 19, 20-033 Lublin, Poland; 3 Independent Researcher, Třebechovická 821, 500 03 Hradec Králové, Czech Republic; macshort@tiscali.cz; 4 Duna-Ipoly National Park Directorate, H-2509 Esztergom, Hungary; szvalent@gmail.com; 5 Independent Researcher, Tršice 370, 783 57 Tršice, Czech Republic; filip.trnka88@gmail.com

**Keywords:** Coleoptera, Curculionidae, Hyperinae, *Hypera libanotidis*, mature larva, pupa, host plant, life history, biology

## Abstract

Last instar larva and pupa of *Hypera libanotidis* Reitter, 1896 (Curculionidae: Hyperini) are described for the first time and compared with 44 other hyperine taxa. Larval morphology generally matches the diagnostic characters of Hyperini but shows distinctive traits, including thorn-like setae on conspicuous black protuberances, relatively long body setae, and dense spiculate coverings in larvae and pupae. Several unusual pupal characters, such as an atypical mesocoxal seta and a peculiar spiracular covering, further distinguish this species. These characters indicate close morphological affinity with species within the subgenus *Eririnomorphus* and also the genus *Metadonus*, suggesting a potentially close phylogenetic relationship. Biological observations confirm typical hyperine traits, including ectophytic larval feeding, cryptic coloration, and cocoon construction prior to pupation. Larval coloration, especially in early instars, partly corresponds with the host plant *Libanotis pyrenaica*. Larvae pupate in mesh-like cocoons on host plant remains, and no larval parasitism was observed. The species is associated with xerothermic loess grasslands, with adults apparently overwintering.

## Simple Summary

The present study provides the first detailed description of the immature stages and biological traits of *Hypera libanotidis*, significantly expanding the current knowledge of this poorly known hyperine species. Larval and pupal morphology largely conforms to the diagnostic characters of Hyperini but also exhibits several distinctive characters, including thorn-like setae borne on dark protuberances, dense spiculate coverings, and unusual pupal structures. These characters support the close morphological affinity of *H. libanotidis* with species of the subgenus *Eririnomorphus* and highlight notable similarities with *Metadonus*, suggesting a potentially close phylogenetic relationship that warrants future formal analysis. Biological observations confirm typical hyperine traits, such as ectophytic larval feeding, cryptic coloration, and cocoon construction prior to pupation. The close association with *Libanotis pyrenaica*, synchronized larval development, and apparent overwintering in the adult stage are consistent with earlier historical records and newly acquired field data. The absence of observed parasitism, despite its prevalence in Hyperini, further underlines the biological distinctiveness of this species. Overall, the detailed documentation of immature stages enables reliable identification of *H. libanotidis* in its larval and pupal phases and underscores the importance of developmental and biological data for taxonomy, phylogeny, and conservation of rare hyperine weevils.

## 1. Introduction

The final phylogenetic position of the Hyperinae within Curculionidae still remains incompletely resolved despite several molecular and morphological studies over the past few decades [1,2]. Recent analyses have consistently placed hyperines within a broader clade that includes Entiminae, Cyclominae, and Gonipterinae, suggesting close evolutionary relationships among these lineages [1,3,4,5,6]. However, the precise delimitation and rank of Hyperinae within this complex remain a subject of ongoing debate. Firstly, Petri [7] provided the first and also last formal morphological definition of the group, based on ten characters, though only those of the trochanters, claws, and pygidium are truly diagnostic, and none are unique to Hyperini [8]. Petri recognized the group as a tribe (not a subfamily) and divided it into two subtribes, Hyperina and Cepurina, using mesepimeral and metanepisternal characters. Currently, Hyperini comprise about 44 genera and 500 described species [9]. Based on distributional and morphological evidence, Skuhrovec and Alonso-Zarazaga [9] provisionally divided the group into three main lineages: (1) the Palaearctic region (Hyperina)—the largest group, with about 370 species and limited representation in the Nearctic (ca. 20); (2) the circumtropical region (Cepurina)—comprising species from the Neotropical (ca. 40), Afrotropical (ca. 16), and Oriental (ca. 2) regions; and (3) the Australo-Pacific Hyperini including *Phaeopholus* Roelofs, 1873—with about 45 species restricted to the Australo-Pacific region.

Recent taxonomic and morphological research on the Hyperinae has focused mainly on the Palaearctic fauna. The most substantial advances have come from the works of Skuhrovec [10,11,12,13] and Skuhrovec and Bogusch [14], who examined the larval morphology of several genera, including *Donus* Jekel, 1865; *Brachypera* Capiomont, 1868; *Hypera* Germar, 1817; and *Metadonus* Capiomont, 1868. These studies, together with subsequent taxonomic revisions [15,16], clarified the complex nomenclature and relationships within these large and taxonomically difficult genera. Other historical and recent taxonomic changes were further summarized in Alonso-Zarazaga et al. [17], and Skuhrovec and Alonso-Zarazaga [9]. Many diagnostic characters currently used for species- and genus-level identification are derived from a limited subset of taxa. As a result, a comprehensive comparative study encompassing representatives of all genera, subgenera, and major species groups of Hyperini is still needed to establish the true diversity and phylogenetic value of both adult and immature morphological traits (see [8,14,17]).

Many weevils live a concealed lifestyle, making their biology and ecology difficult to study, and Hyperinae are no exception. The Hyperinae are distinguished primarily by two biological traits: ectophagous larvae and a distinctive meshed cocoon spun from protein secreted by the Malpighian tubules (see [14]). Their life histories often remain shrouded in mystery, as exemplified by *Hypera libanotidis* Reitter, 1896, a species regarded as a Moravian endemic [18]. The species was first discovered on the limestone hill Kotouč near Štramberk at the end of the 19th century. The larvae and pupae were found on *Libanotis pyrenaica* [19]. According to Fleischer [20], Frank bred adults from pupae collected near Štramberk. Fritsch from Nový Jičín also succeeded in rearing the species, and Purkyně [19] later reported that Fritsch collected several thousand specimens there. The massive collection of larvae and pupae at the turn of the 19th and 20th centuries likely contributed to the depletion of the population and, combined with habitat destruction caused by quarrying of the limestone hill, probably led to the species’ disappearance. All voucher specimens in museum and private collections originate from these early collections [21]. Other reported localities, such as “Uherské Hradiště,” “Bescydi,” and the surroundings of Frýdek-Místek, are uncertain due to missing or dubious labels. Similarly, the record from Slovakia (Devínska Kobyla) is certainly based on a misidentified specimen (*H. arator*) [21]. Recently, Podlussany et al. [22] and Szénási [23] published this mysterious weevil from Hungary. Given the lack of credible records for more than a century and the species’ failure to be rediscovered despite extensive entomological surveys, *Hypera libanotidis* was until today considered extinct in central Europe. Finally, Legalov et al. [24] reported the occurrence of this species (under the combination *Zaslavskypera libanotidis*) in Western Siberia. However, this record may represent a misidentification with another striped species of *Hypera*. The striped species within the subgenus *Eririnomorphus* Capiomont, 1868 require a detailed taxonomic revision (see [25] for details on this group).

The hyperine studies of immature stages and biology by Skuhrovec [10,11,12,13,25,26]; Skuhrovec and Bogusch [14]; and Winkelmann and Skuhrovec [27] have elucidated the biology of most species within this group, demonstrating that research on this group is most efficiently conducted through the examination and collection of larvae, which are considerably easier to locate than the cryptic adults. Building upon this methodological framework, Filip Trnka, based on a new publication by Szénási [23], applied targeted searches on the host plant in Hungary, where he successfully rediscovered this mysterious weevil and obtained new fresh material of immature stages, and some of them bred up to adults. Based on this material, the larva and pupa of *Hypera libanotidis* are described here for the first time, and additional observations on the biology and field behavior of the species in central Europe are provided.

## 2. Materials and Methods

### 2.1. Insect Collection

#### *Hypera libanotidis* Reitter, 1896

**Material examined**: HUNGARY: Veszprém County: Balatonalmádi, Vöröshegy; dry grassland developed on loess at an altitude of approximately 250 m a. s. l.; 7.iv.2024 (8 mature larvae and 5 pupae: 2 ♂ and 3 ♀), all larvae on *Libanotis pyrenaica* leg. & det. F. Trnka, coll. J. Skuhrovec.

### 2.2. Morphological Descriptions

All larval and pupal material was preserved in Pampel’s fixation fluid (see [28]) and subsequently used for morphological descriptions. Slide preparations followed the procedure outlined by May [29]: each larva was decapitated, and the head was cleared in a 10% potassium hydroxide (KOH) solution before being rinsed in distilled water. After clearing, the mouthparts were separated from the head capsule, and both the head capsule and mouthparts were mounted on permanent microscope slides in Euparal. The remaining body parts were mounted on temporary slides in 10% glycerine.

Observations and measurements were performed using light microscopes equipped with calibrated oculars (Olympus BX 40 (Olympus, Tokyo, Japan) and Nikon Eclipse 80i (Nikon, Minato, Tokio Prefecture, Japan). The following larval characters were measured: head width, total body length (larvae fixed in a C-shape were measured segment by segment), and maximum body width (usually at the metathorax or abdominal segments I–IV). For pupae, the total length and maximum width were recorded. The lengths of all setae are provided in the corresponding figures.

Images of details of immature morphology were taken with a HIROX digital microscope (RH-2000) (HIROX, Limonest, France). The larvae and pupae selected for pictures using an SEM (scanning electron microscope) were first dried in absolute ethyl alcohol (99.8%), rinsed in acetone, treated by CPD (critical point drying), and then gold-plated. A TESCAN Vega 3 SEM (Tescan, Brno, Czech Republic) was used for the examination of selected structures.

Images were processed with Adobe Photoshop, Corel Photo-Paint 11, and/or GIMP 2. Setae counts for bilateral structures are given for one side only.

Terminology and abbreviations for larval and pupal setae follow Scherf [30], May [31,32], and Marvaldi [33,34,35].

## 3. Results

### 3.1. Descriptions

#### 3.1.1. Description of Mature Larva

*Measurements* (in mm): Body length: 10.5–11.1 (mean 18.8). The widest place in the body (abdominal segments II–VI) measures up to 3.1. Head width: 0.83–0.93 (mean 0.86).

**Figure 1:**
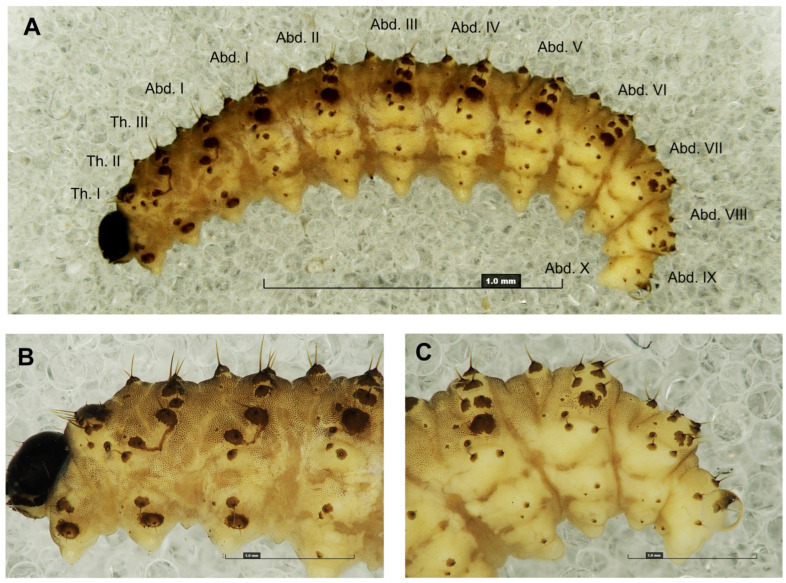
*Hypera libanotidis* Reitter, 1896, mature larva, lateral view: (**A**)—habitus; (**B**,**C**)—magnifications (photos J. Skuhrovec).

**Figure 2:**
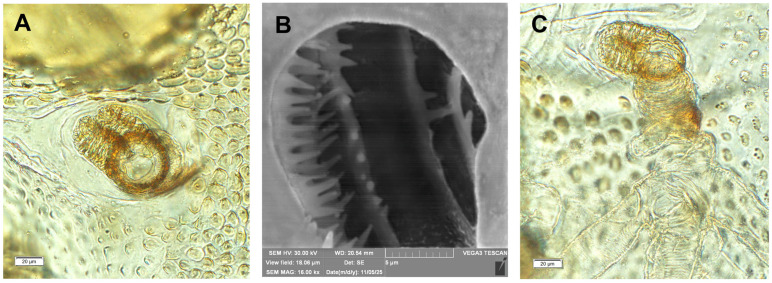
*Hypera libanotidis* Reitter, 1896, mature larva, spiracles: (**A**)—thoracic; (**B**)—inside if the thoracic tube; (**C**)—abdominal (photos R. Gosik).

*Coloration:* Dark brown to black head (Figure 1A,B). All thoracic and abdominal segments yellowish with dark pigments. Dorsal and dorsolateral parts of the body densely covered with dark brown, knobby asperities. Ventral and ventrolateral body part (especially on abdominal segments VI–X) light yellow and less densely covered with asperities. Pronotal sclerite irregular shape, dark pigmented. The protuberances of setae dark pigmented (Figure 1A–C and Figure 2A–C).

**Figure 3:**
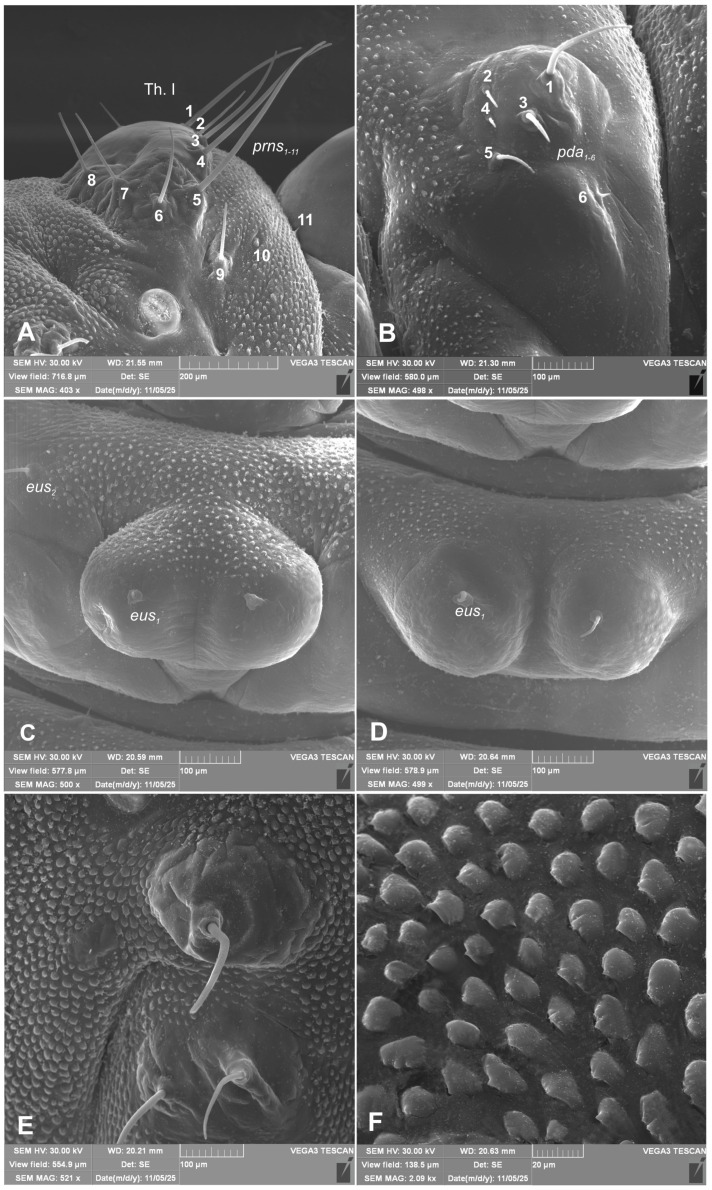
*Hypera libanotidis* Reitter, 1896, mature larva, magnification of selected body parts: (**A**)—pronotum of thoracic segment I; (**B**)—pedal area; (**C**,**D**)—eusternal folds of first and second thoracic segments; (**E**)—spiracular and epipleural folds of thoracic segment II; (**F**)—magnification of cuticle of lateral part of abdominal segment II (Th. I—number of thoracic segment; setae: *eus*—eusternal, *pda*—pedal, and *prns*—pronotal) (photos R. Gosik).

*General*: Body elongated, almost straight (except VIII and IX abdominal segments which are curved to downside) rounded in cross section (Figure 1A–C). All spiracles bicameral, the thoracic placed between the pro- and mesothorax, the abdominal spiracles (8 pairs) located dorsolaterally, close to the anterior margin of abdominal segments I–VIII (Figure 1A–C and Figure 2A–C). Prothorax distinctly smaller than meso- and metathorax, both are equal size. Abdominal segments I–VI of almost equal length, segments VII to IX decreasing gradually to the terminal parts of the body. Abdominal segment X reduced to four anal lobes of unequal size, the dorsal being distinctly the largest, the lateral pair equal in size, and the ventral lobe very small. Anus located terminally; ambulatory ampullae bilobate up to circulate. Abdominal segments I–VIII dorsally divided into two lobes of unequal size; segment IX dorsally undivided. Pedal area, epipleural, pleural and eusternal lobes of abdominal segments well isolated, conical, prominent (Figure 3A–E).

**Figure 4:**
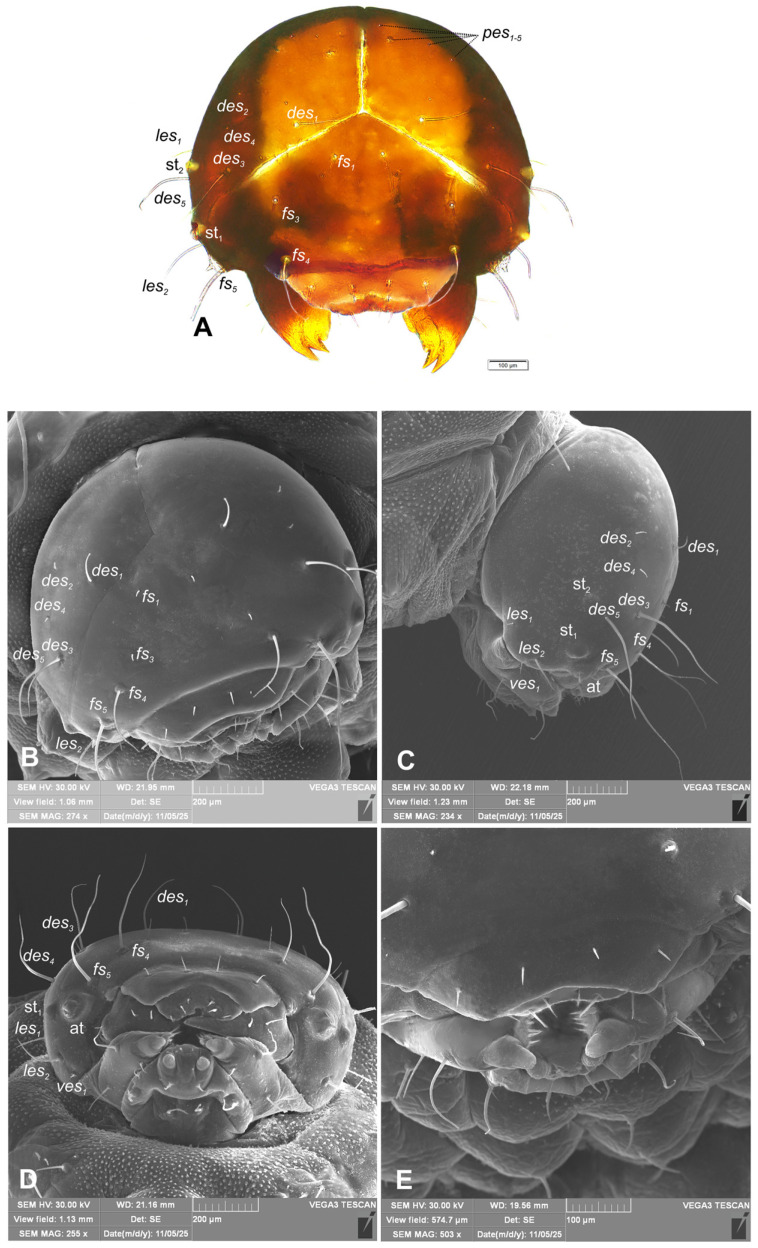
*Hypera libanotidis* Reitter, 1896, mature larva, head: (**A**)—dorsal view (photo); (**B**)—dorsal view (SEM); (**C**)—lateral view (SEM); (**D**)—ventral view (SEM); (**E**)—dorsal view, magnification (SEM) (at—antenna and st—stemmata; setae: *des*—dorsal epicranial, *fs*—frontal epicranial, *les*—lateral epicranial, *pes*—postepicranial, and *ves*—ventroepicranial) (photos R. Gosik).

*Head capsule* (Figure 4A–E): Head sub-oval, wide, endocranial line absent. Frontal sutures on head distinct, extended to antennae. Two stemmata (st), in the form of a dark pigmented spot, both located on each side anterolaterally, close each other. Des_1_ elongated, located in upper part of the central part of epicranium; des_2_ short, near to side of epicranium; des_3_ elongated, located anteriorly near to frontal suture; des_4_ short, located in the central part of epicranium; des_5_ elongated, located anterolaterally (Figure 4A–C). Both fs_1_ and fs_3_ short, placed medially, fs_2_ absent, fs_4_ elongated, located mediolaterally, and very long fs_5_ located anterolaterally, close to the epistoma (Figure 4A–C). Les_1_ medium, les_2_ elongated; and ves_1–2_ short. Epicranial area with 5 postepicranial setae (pes_1–5_) and 4 pores.

**Figure 5:**
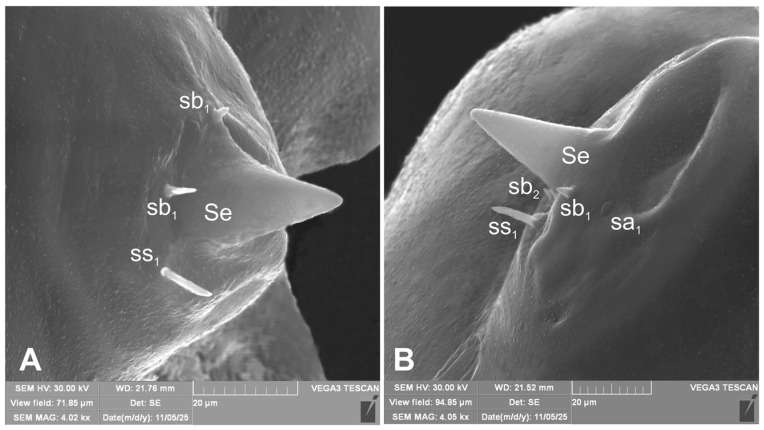
*Hypera libanotidis* Reitter, 1896, mature larva, antenna (SEM): (**A**)—lateral view, right side; (**B**)—lateral view, left side (sa—sensillum ampullaceum, sb—sensillum basiconicum, Se—sensorium, and ss—sensillum styloconicum) (photos R. Gosik).

*Antennae* located at the end of the frontal suture on each side, membranous and slightly convex basal article bearing conical elongated sensorium; basal membranous article with 3 sensillae different in both shape and length: single styloconicum ss, two basiconicum sb and single ampullaceum sa (Figure 5A,B).

**Figure 6:**
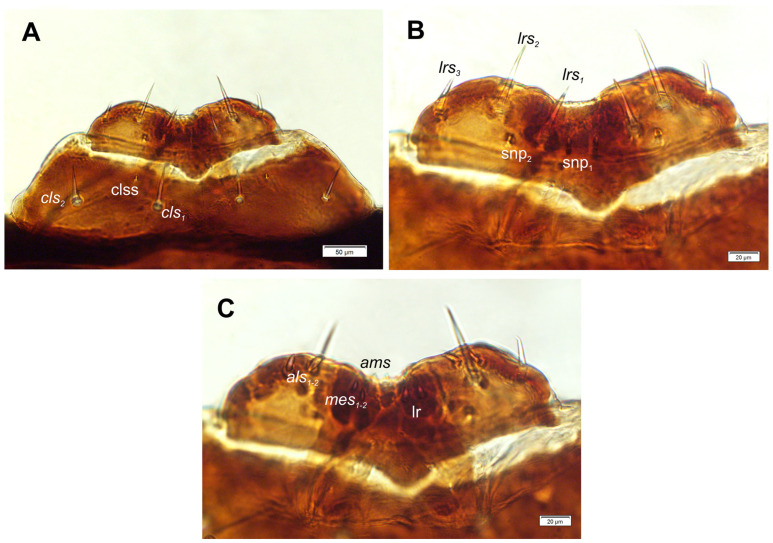
*Hypera libanotidis* Reitter, 1896, mature larva, labrum and clypeus: (**A**)—general view; (**B**)—labrum; (**C**)—epipharynx; (clss—clypeal sensillum, lr—labral rods, snp—sensory pore, setae: *als*—anterolateral, *ams*—anteromedial, *cls*—clypeal, *lrs*—labral, *mes*—median) (photos R. Gosik).

*Clypeus* (Figure 6A) approx. 4 times as wide as long with medium long cls, almost equal in length, localized posterolaterally, and 1 sensillum placed between the setae; anterior margin rounded to the inside.

**Figure 7:**
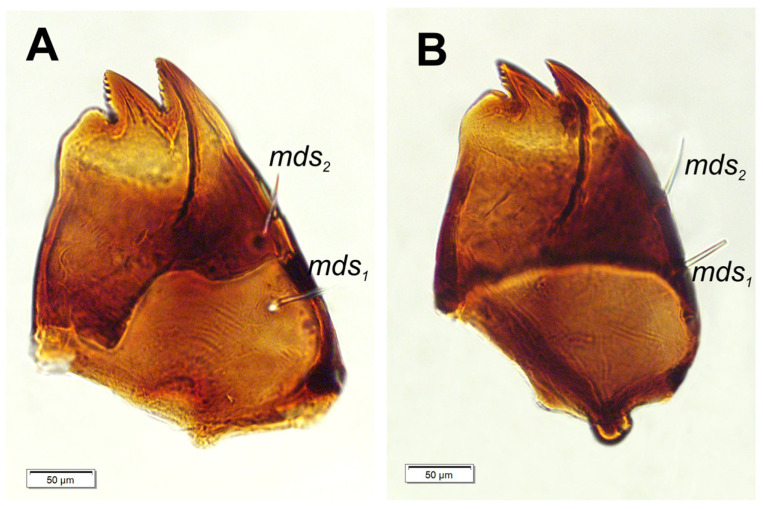
*Hypera libanotidis* Reitter, 1896, mature larva, mouthparts, right mandible: (**A**)—dorsal view; (**B**)—ventral view (setae: *mds*—mandible) (photos R. Gosik).

**Figure 8:**
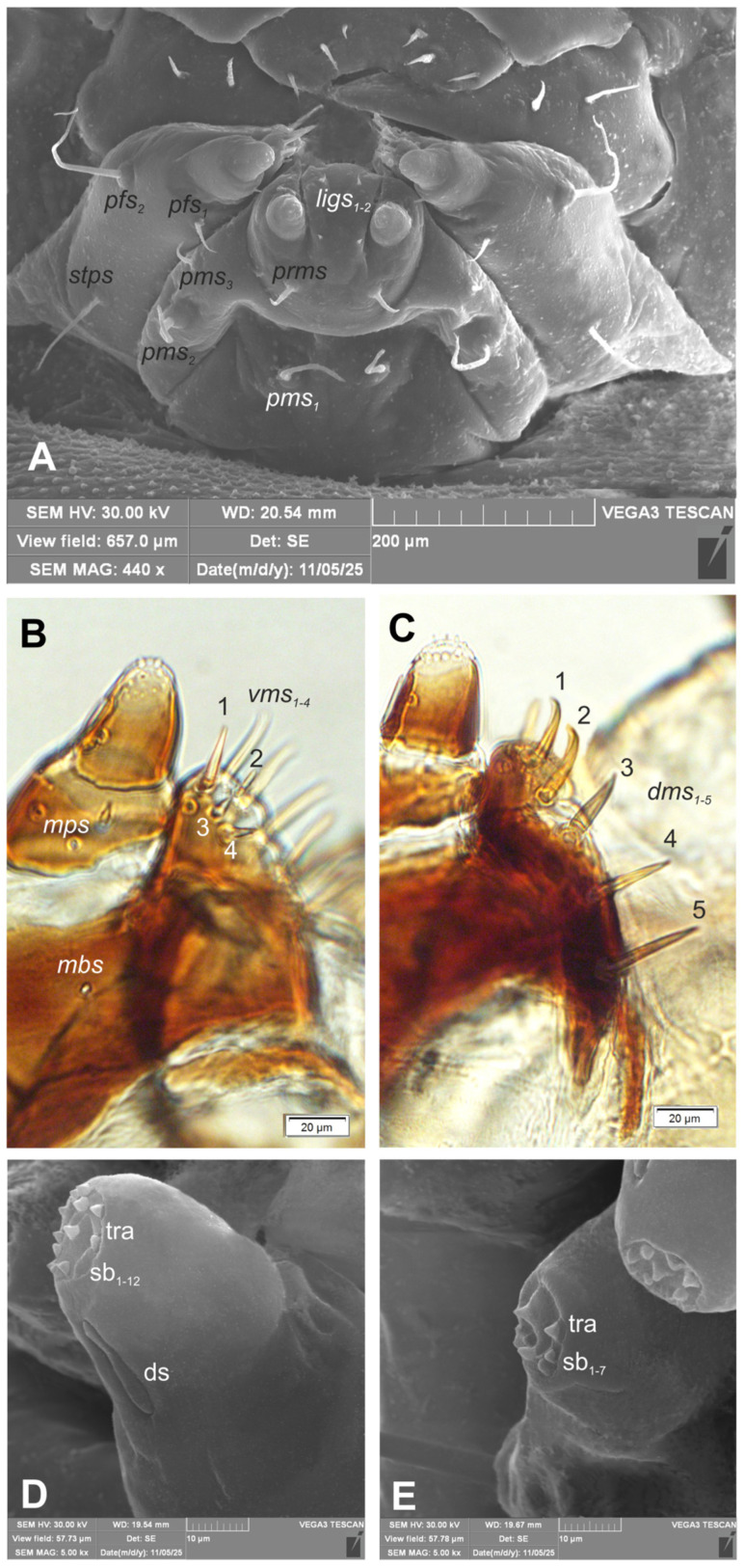
*Hypera libanotidis* Reitter, 1896, mature larva, maxillolabial complex: (**A**)—general view (SEM); (**B**)—maxilla, apical part, ventral view; (**C**)—maxilla, apical part, dorsal view; (**D**)—maxillary palp (SEM); (**E**)—labial palp (SEM) (ds—sensillum digitiform, sb—sensillum basiconicum, and tra—terminal receptive area; setae: *dms*—dorsal malar, *ligs*—ligular, *mbs*—basioventral, *mps*—maxillary palps, *pfs*—palpiferal, *pms*—postmental, *prms*—premental, and *stps*—stipital, *vms*—ventral malar) (photos R. Gosik).

*Mouth parts:* Labrum (Figure 6A) approximately 4.2 times as wide as long, with 3 hairform *lms*: *lms*_1_ medium, placed posteromedially, close to the margin with clypeus, *lms*_2_ elongated, located anteromedially and *lms*_3_ short, located anterolaterally; anterior margin double sinuate. Epipharynx (Figure 6C) with 2 very short, digitate *als*, almost equal in length; 1 very short digitate *ams*; and 2 short, digitate *mes*, and 2 sensoric pores snp; labral rods (lr) sub-oval, irregular, apical part more sclerotized. Mandibles (Figure 7A,B) distinctly broad, trifid, tooth of unequal height; slightly truncate; first tooth wide, blunt, second and third sharp, triangular, slightly curved, with serrated inner edges. Both *mds* hairform, relatively long, located in distinct holes. Maxilla (Figure 8A–E) stipes with 1 elongated *stps*, medium *pfs*_1_, elongated *pfs*_2_ and minute 1 *mbs*; mala with 5 bacilliform *dms*, almost equal in length; 4 *vms* various in length (1 medium and 3 min); *vms* distinctly shorter than *dms* (Figure 8B,C) Maxillary palpi with two palpomeres; basal palpomere with 1 very short *mps* and two sensilla; length ratio of basal and distal palpomeres: 1:0.8; distal palpomere with one digitiform sensillum and 12 sensillae basiconicum on terminal receptive area tra. Praelabium (Figure 8A) almost rounded, with 1 relatively long *prms*; ligula with sinuate margin and 2 hairform very short to minute *ligs*, unequal in length; premental sclerite well visible, ring shaped. Labial palpi one-segmented; with 7 sensillae basiconicum on terminal receptive area tra (Figure 8D,E). Postlabium (Figure 8A) with 3 *pms*: long *pms*_1_ located anteriorly, very long *pms*_2_ placed mediolaterally, and medium *pms*_3_ placed anterolaterally; surface of postlabium partly covered by knobby cuticular processes.

**Figure 9:**
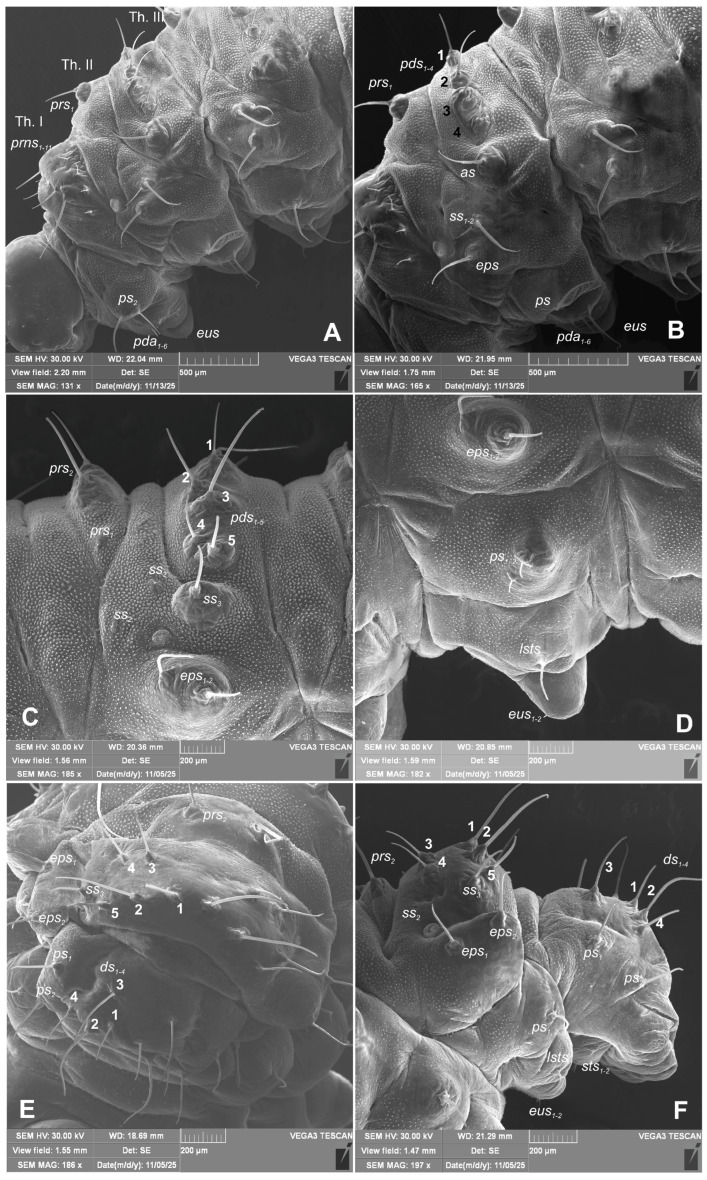
*Hypera libanotidis* Reitter, 1896, mature larva, habitus: (**A**,**B**)—lateral view of thoracic segments; (**C**,**D**)—lateral view of abdominal segment III; (**E**)—dorsal view of abdominal segments VII–IX; (**F**)—lateral view of abdominal segments VIII–X (Th. I–III—number of thoracic segment; setae: *as*—alar, *ds*—dorsal, *eps*—epipleural, *eus*—eusternal, *pda*—pedal, *pds*—postdorsal, *prns*—pronotal, *prs*—prodorsal, *ss*—spiracular, *ps*—pleural, and *sts*—sternal) (photos R. Gosik).

*Chaetotaxy of body:* All setae hair-like, various in length (minute, medium to elongated) placed on conical protuberances (Figure 1A–C). **Thorax**: Prothorax (Figure 9A–F) with 11 various in length *prns* of which setae 1, 3, 4, are long, 5 very long, 2, 6–9 medium, 10 and 11 min; setae from 1 to 8 placed on premental sclerite, setae 9–11 separately, below premental sclerite; 2 elongated *ps* and 1 min *eus*. Mesothorax and metathorax each (Figure 9A,B) with 1 min and 1 elongated *prs*; 4 elongated *pds*; 2 elongated *as*; 2 medium and 1 min *ss*; 1 medium *eps*; 2 min and 1 elongated *ps*; and 1 min and 1 short *eus*. Each pedal area of thoracic segments well separated, with 6 various in length *pda*, 5 of them on pigmented pedal area, last one on separated conical protuberance (Figure 2B and Figure 9A,B). **Abdomen**: Abdominal segments I–VII (Figure 9C–F) with 1 min and 1 elongated *prs*; 5 medium to elongated *pds*; 2 min and 1 medium *ss* (segment VIII with 1 min and 1 elongated *ss*); 2 medium *eps* of almost equal length; 2 short *ps* of equal length; 1 medium *lsts*; and 1 min and 1 short *eus*. Abdominal segment IX (Figure 9E,F) with 4 elongated *ds*; 2 medium *ls*; and 1 short and 1 medium *sts*. Abdominal segment X without setae.

#### 3.1.2. Description of Pupa

*Measurements* (in mm): Body length (♀, ♂): 5.6–6.0; at the widest region: 2.9–3.4. The widest place in the body is commonly between the apex of the meso- or metafemora.

**Figure 10:**
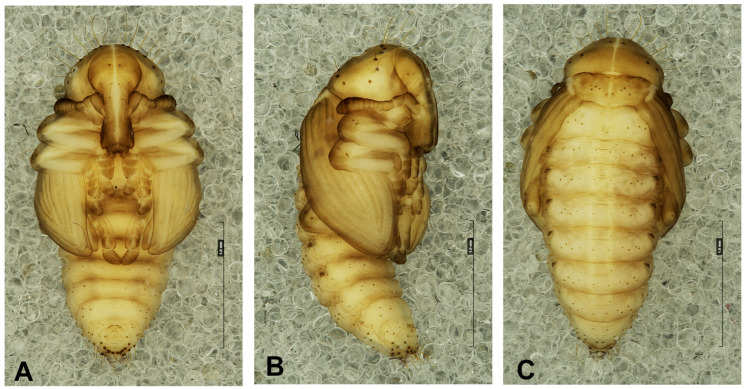
*Hypera libanotidis* Reitter, 1896, pupa: (**A**)—ventral view; (**B**)—lateral view; (**C**)—dorsal view (photos J. Skuhrovec).

*Coloration:* Body yellowish, spots around setae dark brown pigmented (Figure 10A–C).

**Figure 11:**
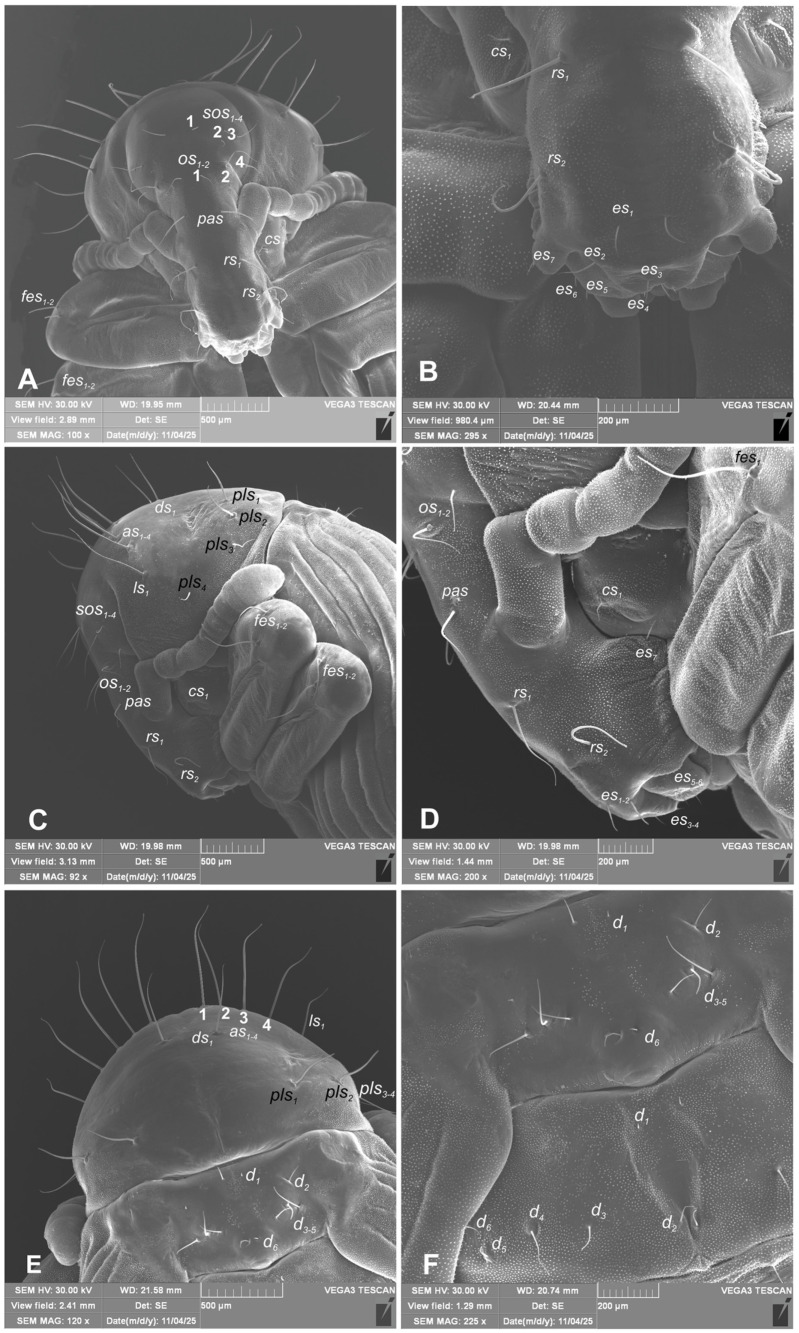
*Hypera libanotidis* Reitter, 1896, pupa: (**A**)—head and rostrum; (**B**)—magnification of terminal part of rostrum; (**C**)—lateral view of head, rostrum and pronotum head and rostrum; (**D**)—magnification of rostrum, lateral view; (**E**)—dorsal part of pronotum and mesonotum; (**F**)—dorsal view of meso- and metanotum (setae: *as*—apical, *cs*—coxae, *d*—dorsal, *ds*—discal, *es*—epistomal, *fes*—femoral, *os*—orbital, *pls*—posterolateral, *rs*—rostral, and *sos*—superorbital) (photos R. Gosik).

**Figure 12:**
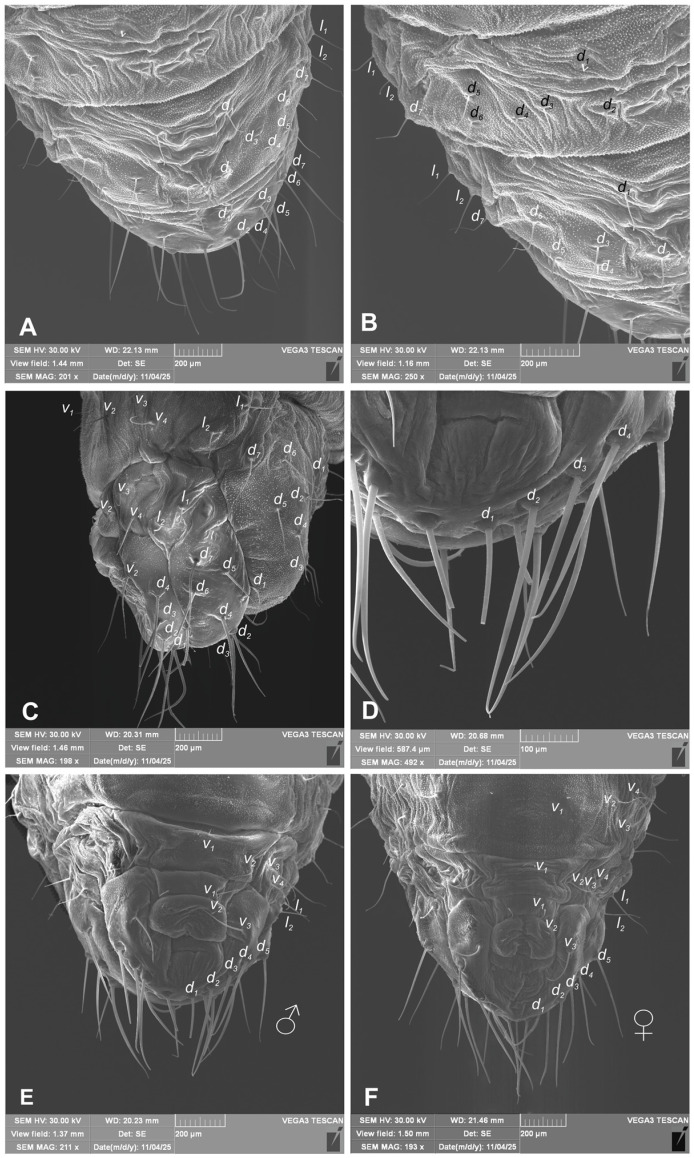
*Hypera libanotidis* Reitter, 1896, pupa: (**A**)—dorsal part of last abdominal segments; (**B**)—dorsal part of last abdominal segments, magnification; (**C**)—lateral part of last abdominal segments; (**D**)—ventral part of last abdominal segments, magnification; (**E**)—ventral part of last abdominal segments, male; (**F**)—ventral part of last abdominal segments, female (setae: *d*—dorsal, *l*—lateral, and *v*—ventral) (photos R. Gosik).

**Figure 13:**
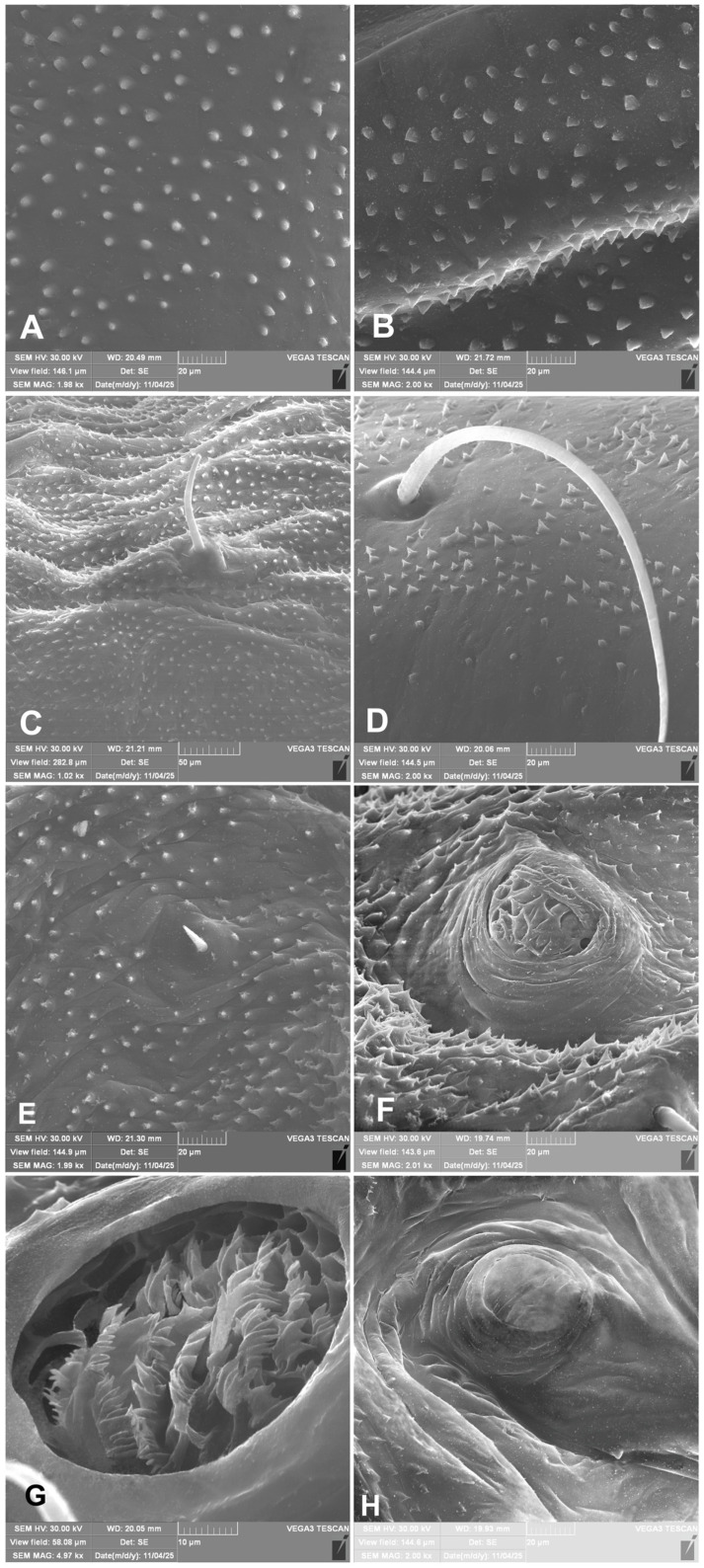
*Hypera libanotidis* Reitter, 1896, pupa, magnification of selected body parts: (**A**)—cuticle, prodorsum; (**B**)—cuticle, elytra; (**C**–**E**)—cuticle abdomen; (**F**)—closed functional spiracle of third abdominal segment; (**G**)—opened functional spiracle of second abdominal segment; (**H**)—atrophied spiracle of seventh abdominal segment (photos R. Gosik).

*Morphology* (Figure 10, Figure 11 and Figure 12): Body rather elongated, only slightly curved. Rostrum relatively long, approximately 2.5 times as long as wide, extended to metacoxae. Antennae relatively long and stout. Pronotum 1.75 as wide as long. Mesonotum and metanotum of almost equal length. Abdominal segments I–III equally in length, segments IV-VI diminish gradually to the end of the body, segment VII semicircular, segments VIII and IX distinctly smaller than other abdominal segments. Gonotheca (abdominal segment IX) in females divided, in male undivided (Figure 11E,F). Cuticle sparsely covered with fine asperities or smooth (Figure 12A–E). Spiracles on lateral parts of abdominal segments I-V function, on segment VI atrophied, on next abdominal segments invisible (Figure 13F–H).

*Chaetotaxy* (Figure 11A–F and Figure 12A–F): Setae well visible, hair-like, elongated to short, yellow or transparent. Head capsule includes only 4 *sos* various in length, 1 medium *os* and 1 medium *pas*. Rostrum with 2 medium *rs*, equal in size. Epistoma with 7 min setae *es*. Pronotum with 4 elongated *as*, 1 medium *ds*, medium 2 *ls* and 3 medium *pls*. Dorsal parts of mesothorax with 1 short seta located posteromedially, 3 short to medium seta posterolaterally and 1 short seta located along its anterior margin. Dorsal parts of metathorax with 1 min seta located along its anterior margin and next 5 short setae along its posterior margin. Each apex of femora with 2 elongated *fes*. Procoxa with short *cs* (Figure 11A–F). Abdominal segments I–VIII each with 1 seta located posteromedially (*d*_1_) and 5 setae (*d*_2–6_) located along their anterior margins; setae elongated, hair-like. Abdominal segment IX with 4 elongate setae *d*_1–4_ along its anterior margin and 2 short and 1 medium ventral setae. Dorsal setae on segments VIII and IX distinctly longer than other abdominal setae. Abdominal segments V–VIII with 2 medium lateral setae, and 2 short and 2 medium ventral setae. Urogomphia absent.

### 3.2. Biological Observations

**Figure 14:**
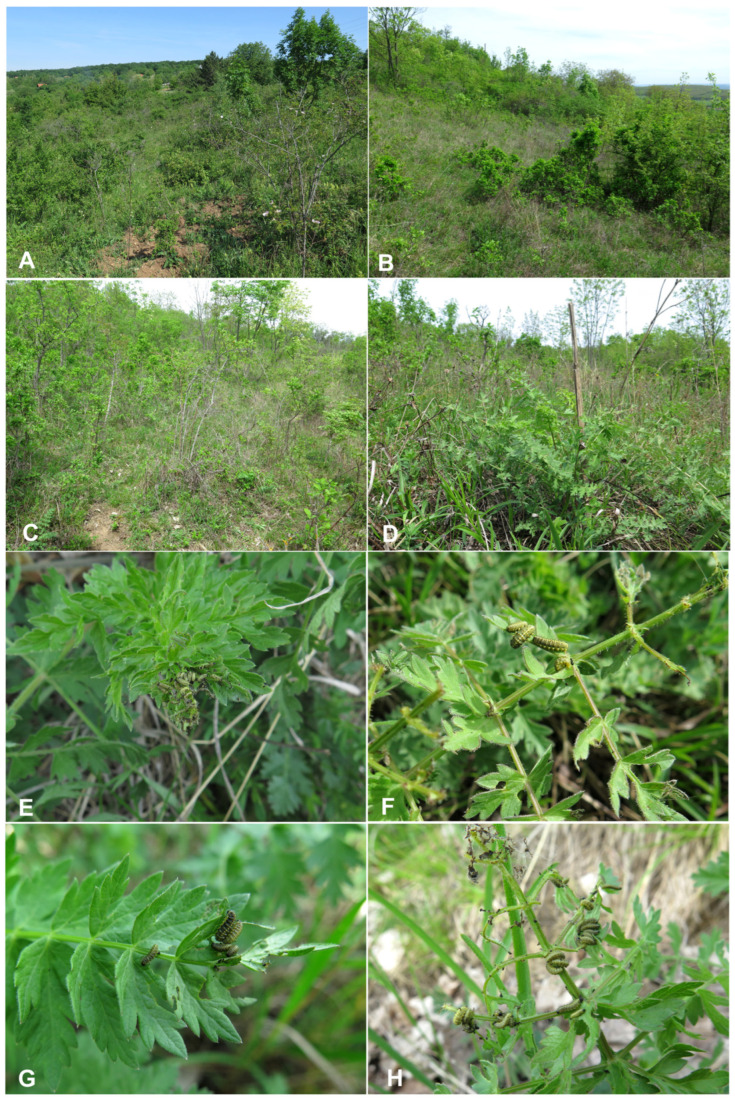
*Hypera libanotidis* Reitter, 1896: (**A**–**D**)—habitats; (**E**–**H**)—various instars of larvae feeding on host plant *Libanotis pyrenaica* (photos F. Trnka).

*Habitats*: *Hypera libanotidis* occurs in xerothermic grasslands developed on loess substrates, characterized by advanced succession and a high degree of overgrowth by shrubs and scattered trees (locality Vöröshegy, Figure 14A,B). At both studied localities, the habitat supported a rich population of the host plant. During a repeated visit on 11 May 2024, ten last-instar larvae were found on host plants; all larvae occurred exclusively on plants growing in shade or partial shade beneath bushes, indicating a preference for sheltered microhabitats within otherwise open dry grassland.

*Adult behavior*: Adults of *Hypera libanotidis* (Figure 14H) are extremely difficult to observe in the field. During larval sampling, a single adult was collected by one of the co-authors (Jiří Krátký) during daytime; the specimen was found in the soil beneath the host plant and appeared to be in a state of aestivation shortly after emergence (7 April 2024, Pécsely, Öreg-hegy). Despite the assumption of predominantly nocturnal activity, only one adult specimen was recorded during targeted searches at the same locality on 11 May 2024.

According to long-term field observations (Valentin, pers. observ.), adults can be observed from the winter months onwards, occasionally even beneath snow cover, which is now increasingly rare. The earliest recorded observation dates back to 2 January, with records becoming more frequent in February. These data strongly suggest that the species overwinters in the adult stage. During the cold season, specimens remain inactive and occur mainly in dense grass litter in close proximity to the host plant, often aggregated around its basal rosette.

From March onwards, adult activity increases with rising temperatures. On warm days, particularly at temperatures around 20 °C, adults are frequently observed running on the ground or feeding and copulating on the leaves of the host plant. During cooler afternoon and evening hours, specimens retreat again to the basal leaf rosette, in some cases in close association with *Hypera striata* individuals.

Adult activity generally ceases by the end of April, after which only dead specimens or fragmentary remains are found. In August, isolated elytra with characteristic patterning can occasionally be detected in the habitat, indicating the presence of the species earlier in the season.

*Host plant*: Larvae of *Hypera libanotidis* of all instars were observed aggregated on the basal rosettes of the host plant (Figure 14A–H), frequently concentrated on the apical part of a single leaf (Figure 14A–H). First- and second-instar larvae were most commonly found in close proximity to each other, forming small feeding groups (Figure 14A–H), whereas mature instar larvae were also present but tended to disperse over the plant and occupy additional leaves (Figure 14A–H). Larval activity was strictly diurnal. Larval feeding resulted in characteristic damage to the host plant leaves, with the leaf tissue typically consumed from the apical portion towards approximately the middle of the leaf blade.

**Figure 15:**
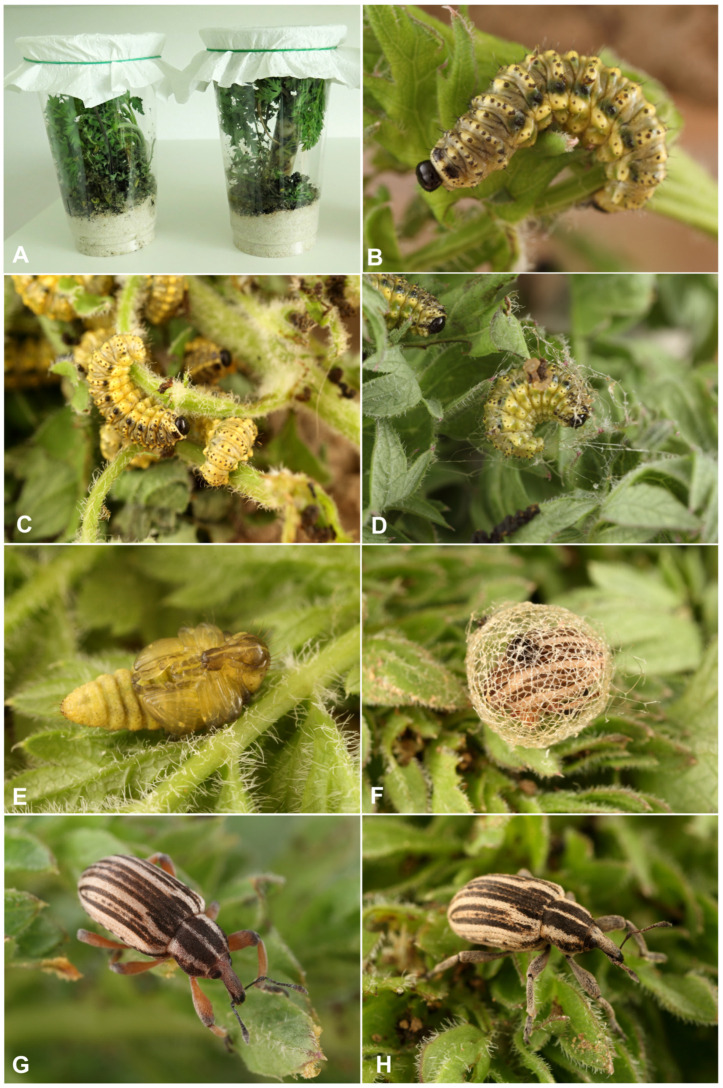
*Hypera libanotidis* Reitter, 1896: (**A**)—laboratory breeding; (**B**,**C**)—mature larvae feeding on host plant *Libanotis pyrenaica*; (**D**)—larva forming cocoon; (**E**)—pupa; (**F**)—fresh imago in cocoon; (**G**,**H**)—adult (photos F. Trnka).

*Life cycle*: At the Vöröshegy locality, approximately 100 larvae of all instars were collected and rearing commenced on 7 April 2024. The first pupae appeared within several days (Figure 15A–H), and all surviving larvae completed pupation within a two-week period. Of the collected larvae, 95 successfully pupated, while the remaining individuals died for unknown reasons, most likely during ecdysis between larval instars. The emerged adults were subsequently maintained and regularly supplied with fresh host plant material. Adult activity in captivity was low; specimens spent most of their time concealed and exhibited limited movement, leaving their shelters primarily to feed or imbibe moisture. Approximately one-third of the reared adults survived in laboratory conditions until early September. These observations suggest that, under natural conditions, adults probably enter aestivation shortly after emergence and overwinter as adults, with mating occurring in early spring.

Pupal development under laboratory conditions (Figure 15A–H) was noticeably faster than expected under natural conditions (Skuhrovec, pers. observ.). This acceleration can likely be attributed to two main factors. First, the rearing was conducted under stable temperature conditions, allowing for continuous development without thermal interruptions. In contrast, in natural environments larval development is exposed to daily temperature fluctuations, particularly nocturnal cooling, which generally prolongs developmental time. Second, the provision of fine sawdust in the rearing containers appeared to facilitate pupation. Mature larvae readily incorporated the sawdust into their cocoons, reducing the need to construct entirely self-produced protective structures. This likely lowered energetic costs associated with cocoon formation and further contributed to a shorter pupal development period compared to larvae developing under natural conditions.

A revisit to the Vöröshegy site on 27 April 2025 revealed the presence of numerous first-instar larvae, whereas second- and mature instar larvae were scarce, indicating a temporally synchronized larval development. Based on these data, mating and oviposition most likely take place during February and March. Under laboratory conditions, pupation occurred predominantly on dry plant material or remains of the host plant and less frequently on the soil surface or on the walls of the rearing containers.

*Biotic interactions:* No parasitism was recorded in the examined larval material. None of the collected larvae showed signs of parasitoid attack, which is in contrast to observations commonly reported for members of the subfamily Hyperinae [14].

## 4. Discussion

### 4.1. Comparison with Immature Stages of Other Hyperini

To date, the larvae of 44 hyperine taxa are known [10,11,13,14,30,32,35,36,37,38,39,40,41,42,43,44,45,46], and detailed descriptions of pupae are available for only 10 taxa [14,37,38,39,40,46,47]. The morphological characteristics of larvae of the tribe Hyperini were primarily defined by Lee and Morimoto [43], May [32], and Marvaldi [36], including epipharynx and maxilla with simple setae, the third dorsal seta (*des*_3_) on the epicranium, the fifth frontal seta (*fs*_5_) longer than the fourth one (*fs*_4_), one-segmented labial palpus, mandible with sharp teeth, indistinct labral rods, present postoccipital condyles, pedal areas that are swollen to form prolegs or large lobes, head maculate and pigmented body. A comparative summary of all recent data was provided by Oberprieler et al. [8]. Later, Nazarenko [48] described and discussed the epipharyngeal morphology of seven hyperine species. His final proposed epipharyngeal formula (comprising three pairs of *als*, one (or two) pairs of *ams*, and two pairs of *mes*) does not correspond at all to the morphology observed in *Hypera libanotidis* (Figure 6A–C). The exact number of certain epipharyngeal setae in weevils, particularly the *ams* and *mes*, remains still unresolved. Marvaldi [33,34] considered the standard weevil epipharynx to have two *ams* and three *mes*; however, when the distal *mes* are located very close to the anterior margin, they may be mistaken for *ams*. This has led to different interpretations even within the same groups (e.g., Tychiini: Skuhrovec et al. [49,50]).

*Hypera libanotidis* have a unique characteristic in that all setae on the larval body are thorn-like and located on distinct black protuberances (Figure 1A–C). This character is shared by four other species of the subgenus *Eririnomorphus* Capiomont, 1868, whose larvae are known [11,12,36]. Comparable larval morphology has also been reported in *Hypera arator* (Linnaeus, 1758) of the subgenus *Kippenbergia* Alonso-Zarazaga, 2005 [10] and in both known *Metadonus* species [14,40]. The subgenus *Eririnomorphus* shares several additional traits with *Metadonus*, extending beyond the presence of specialized setae on raised protuberances (Skuhrovec, unpubl. data). Adults of both groups exhibit notably similar body-scale morphology (see [15,25]) and some of them are associated with comparably harsh or extreme habitats [16,25]. In these respects, species of *Eririnomorphus* resemble *Metadonus* more closely than any other members of the genus *Hypera* [14]. Taken together, these observations point toward a potentially close phylogenetic affiliation between the two groups, a possibility that has yet to be evaluated through a formal phylogenetic analysis of the hyperines.

In comparison with other *Eririnomorphus* and *Metadonus* species, *H. libanotidis* is characterized by relatively long setae on the body, resembling the condition observed in *Hypera rumicis* (Linnaeus, 1758) and contrasting with the short setae found in *H. arundinis* (Paykull, 1792) and *Metadonus vuillefroyanus* (Capiomont, 1868). Furthermore, the meso- and metathorax each bear three *ss*, a characteristic shared with *H. arundinis* but differing from *H. rumicis*, which exhibits a different setal arrangement (for more, see keys in [13,14]). The larva and pupa of *H. libanotidis* are likewise distinctive through other characters, e.g., being densely covered with robust spicules rather than typical setae (Figure 1A–C); these are particularly well developed on the elytral sheaths on pupa. A comparable but distinctly different spiculate covering was described by Skuhrovec et al. [51] on larva of the weevil *Eucoeliodes mirabilis*. An additional unusual character of the pupa is the presence of an atypical mesocoxal seta (Figure 11A,D), combined with a still poorly understood, seemingly continuous covering of spiracula (Figure 13F–H), the functional significance of which remains unknown.

Knowledge of the immature stages and life histories of species can help to protect endangered species more effectively. The detailed description of larvae and pupae and their comparisons with known descriptions as reported here demonstrates that it is possible to identify this species in its immature stages, as has been accomplished for other groups (i.e., Entiminae: [52,53,54,55]; Curculioninae, Tychiini: [49,50,56]; Lixinae: [28,57,58,59,60,61]). This process is particularly valuable for rare and endangered species because finding larvae is typically much simpler than finding adults. Additional detailed descriptions for hyperines, precise keys, detailed generic studies and comparisons of all groups could help in future with the completion of a phylogenetic analysis of this group. It could also be very useful in different entomological fields, such as agriculture, biological control, and protection of endangered species.

### 4.2. Biological Specificity

Hyperinae are characterized by two biologically distinctive characters that are otherwise uncommon among Curculionidae: externally feeding (ectophytic) larvae with cryptic coloration and the ability to construct mesh-like cocoons prior to pupation. Both traits were confirmed in the mysterious species *Hypera libanotidis*, albeit with several species-specific characteristics.

Early biological observations on *H. libanotidis*, particularly its close association with *Libanotis pyrenaica*, were provided by Fleischer [20] and Purkyně [19] from primary findings by Fritsch. These historical data fully correspond with the results of the present study. More recent records of adult specimens from Hungary were published by Podlussány et al. [22] and Szénási [23], and additional observations from xerothermic loess grasslands in Hungary were made by three of the present authors (FT, JK, and VS).

Purkyně [19] briefly also characterized larval coloration as generally similar to that of other Hyperini larvae (see Description and Figure 1A–C and Figure 13G,H). Our observations confirm this assessment but allow for a more detailed characterization, primarily owing to broader comparative experience with Hyperini larvae. The larval coloration of younger instars partly matches the surface of the host plant leaves, supporting the hypothesis that cryptic coloration functions as an effective defensive strategy. A similar correspondence between larval coloration and host plant leaves was suggested for *Metadonus vuillefroyanus* by Skuhrovec and Bogusch [14], although this phenomenon was not examined in detail. Cryptic coloration is one of the most widespread anti-predator strategies in insects [62], and in Hyperini it may be particularly important because their larvae feed externally on exposed plant surfaces [14].

We also confirmed that larvae of *H. libanotidis* construct cocoons prior to pupation, as is typical for most species of *Hypera*. Notable exceptions exist within Hyperini, such as *Metadonus vuillefroyanus*, whose larvae pupate in the soil, forming a two-layered cocoon: an inner protective layer surrounding the pupa and an outer layer covered with soil particles [14].

All larval instars of *Hypera libanotidis* were consistently observed in aggregated distributions on the host plant (see Biological Observations). Such spatial clustering of larvae, particularly at early developmental stages, likely increases the frequency of direct interactions among conspecifics and may represent a precondition for intraspecific aggression. Under conditions of high larval density on individual host plants, these interactions can escalate into aggressive behavior, including cannibalism, a phenomenon previously reported in Hyperini larvae [47,63]. This interpretation is further supported by observations of older instar larvae, which were no longer in such close spatial contact and were more widely distributed across the host plant, suggesting a tendency toward dispersal with increasing larval size and mobility.

Finally, none of the larvae examined in this study showed any signs of parasitism. This finding is noteworthy, as the majority of Hyperini species are commonly parasitized by braconid wasps (Skuhrovec, pers. observ.).

## Data Availability

The original contributions presented in this study are included in the article. Further inquiries can be directed to the corresponding author.
